# Recent progress of surface-enhanced Raman spectroscopy for subcellular compartment analysis

**DOI:** 10.7150/thno.56409

**Published:** 2021-03-04

**Authors:** Yanting Shen, Jing Yue, Weiqing Xu, Shuping Xu

**Affiliations:** 1 State Key Laboratory of Supramolecular Structure and Materials, Institute of Theoretical Chemistry, College of Chemistry, Jilin University, Changchun 130012, People's Republic of China.; 2 School of Pharmaceutical Sciences, Key Laboratory of Innovative Drug Development and Evaluation, Hebei Medical University, Shijiazhuang, 050017, China.; 3 Department of Molecular Sciences, ARC Centre of Excellence for Nanoscale BioPhotonics (CNBP), Macquarie University, Sydney, New South Wales 2109, Australia.

**Keywords:** organelles, SERS, label, label-free, subcellular analysis

## Abstract

Organelles are involved in many cell life activities, and their metabolic or functional disorders are closely related to apoptosis, neurodegenerative diseases, cardiovascular diseases, and the development and metastasis of cancers. The explorations of subcellular structures, microenvironments, and their abnormal conditions are conducive to a deeper understanding of many pathological mechanisms, which are expected to achieve the early diagnosis and the effective therapy of diseases. Organelles are also the targeted locations of drugs, and they play significant roles in many targeting therapeutic strategies. Surface-enhanced Raman spectroscopy (SERS) is a powerful analytical tool that can provide the molecular fingerprint information of subcellular compartments and the real-time cellular dynamics in a non-invasive and non-destructive way. This review aims to summarize the recent advances of SERS studies on subcellular compartments, including five parts. The introductions of SERS and subcellular compartments are given. SERS is promising in subcellular compartment studies due to its molecular specificity and high sensitivity, and both of which highly match the high demands of cellular/subcellular investigations. Intracellular SERS is mainly cataloged as the labeling and label-free methods. For subcellular targeted detections and therapies, how to internalize plasmonic nanoparticles or nanostructure in the target locations is a key point. The subcellular compartment SERS detections, SERS measurements of isolated organelles, investigations of therapeutic mechanisms from subcellular compartments and microenvironments, and integration of SERS diagnosis and treatment are sequentially presented. A perspective view of the subcellular SERS studies is discussed from six aspects. This review provides a comprehensive overview of SERS applications in subcellular compartment researches, which will be a useful reference for designing the SERS-involved therapeutic systems.

## Introduction

### Subcellular compartments

Subcellular compartments, including cell membrane, nucleus, mitochondrion, lysosome, and endoplasmic reticulum (ER), etc., are the essential functional subunits within eukaryote cells 1, 2. Each of them plays a specific and indispensable role in supporting the normal functions of cells. As the control center of the cell, the nucleus plays prominent roles in cell proliferation, growth, differentiation, metabolism, and apoptosis 3. Mitochondrion, the “powerhouse” of cells, its primary task is to generate adenosine triphosphate (ATP) and the necessary metabolites to fulfill the survival demands of the cell 4. The lysosome is an acidic subcellular compartment containing numerous hydrolytic enzymes, such as proteases and nucleases. Moreover, lysosome is also involved in various physiological processes, such as cholesterol homeostasis, plasma membrane repair, secretion, cell death, and cell signaling 5, 6.

Given the essential roles of these cellular compartments in cellular activities, it can be speculated that any dysfunction or disruption of the structures and functions of subcellular compartments can result in abnormal intracellular homeostasis and various cellular disorders. For example, disturbances to the nucleus may lead to the abnormal regulation of cell activity and even cause cell death. Various diseases, such as cancers, heart disorders, and brain disorders, are also related to nuclear dysfunction 7. The deficiency of mitochondrion directly affects the energy supply and leads cells to die. Moreover, cell survival and aging are also related to the function of the mitochondrion. Its abnormal function would accompany neurodegenerative diseases such as Alzheimer's disease and multiple sclerosis 8, 9. Many cell functions are related to the lysosome. It is unsurprising that lysosomal dysfunction causes many diseases 10, 11, especially the lysosomal storage disease, one of the most severe diseases that can cause secondary changes, such as autophagy dysfunction, mitochondrial dysfunction, and inflammation. Therefore, the precise and accurate detections of the structures, functions, and microenvironments of organelles are of great significance for deeply understanding their roles in cell life activities and the diagnosis and treatment of diseases.

Besides, targeting therapeutic agents at specific subcellular organelles is the key to maximize the therapeutic index, completely eradicate tumors, and prevent invasion, metastasis, and recurrence. In the context of cancer therapy, the biggest bottleneck is how to deliver the drugs to the lesion locations, since the simple transportations of therapeutic agents into the tumors or cancer cells are not efficient and enough to yield the expected therapeutic outcome 12. During chemotherapy 13, 14, reactive oxygen species (ROS) generated in the therapeutic processes are suggested to be close to these bioactive substances, so that the therapeutic effect and the weak side effect can be ensured 15.

At present, the organelle-related analyses mainly focus on two aspects: (1) the detections of structures and compositions of organelles, and (2) the explorations of microenvironments of organelles (e.g., pH, ROS, reactive nitrogen, and ATP, etc.) and the dynamic changes of their structures and microenvironments during therapeutic processes. Traditional subcellular compartment analytical methods are protein immunoblotting 16, 17 and gel electrophoresis 18, 19, in which the cellular components need to be extracted for further analyses. Mass spectroscopy and deoxyribonucleic acid (DNA) sequencing-based genomics spectrometry 20 are widely applied for the quantitative determinations of specific elements. Besides these methods, many *in situ* characterization techniques have been involved due to the merits of monitoring the dynamic changes of a living cell. Most of them are microscopic techniques since the cells and bacteria are in a micrometer size range. Established microscopic technologies, e.g., electron microscope 21, atomic force microscope 22, and optical technologies, e.g., super-resolution fluorescence microscopy 23 and surface-enhanced Raman spectroscopy (SERS) 24, have been widely applied.

### SERS

Raman spectroscopy is one kind of vibrational spectroscopies, which can provide native and rich chemical information of cellular compositions, including nucleic acids, proteins, and lipids. Compared to broad fluorescence bands, Raman bands are narrow and easy to identify. Besides, water has fewer impacts on the Raman spectra. Therefore, compared to infrared spectroscopy, Raman spectroscopy is more suitable for the detections of biological systems.

However, Raman signals are too weak to be detected because only one Raman photon can be excited out of 10^7^ incident photons, limiting its application in biofields. One of the remarkable achievements in the developing history of Raman scattering is the discovery of SERS, first reported by Fleishmann et al. in 1974 25. This technique can amplify the Raman signal of molecules by 4 to 11 orders 26. It gains ultrahigh sensitivity even down to the single-molecule level under the strong electromagnetic enhancement of plasmonic nanomaterials 27, which endows SERS a high detection sensitivity comparable to fluorescence no matter in sensing or imaging. Besides many improvements in the SERS detection sensitivity, SERS substrates make significant progress as well. They start from the silver electrodes to diverse metal nanostructures in the recent forty years. With the continuous efforts on metal nanostructures/nanoparticles (NPs) in preparation, surface modification, assembly, and grafting technologies within near twenty years, the SERS-active nanomaterials have become flexible, adjustable, and designable, which promote SERS advances in bio and medical applications 28-31.

**Scheme 1:**
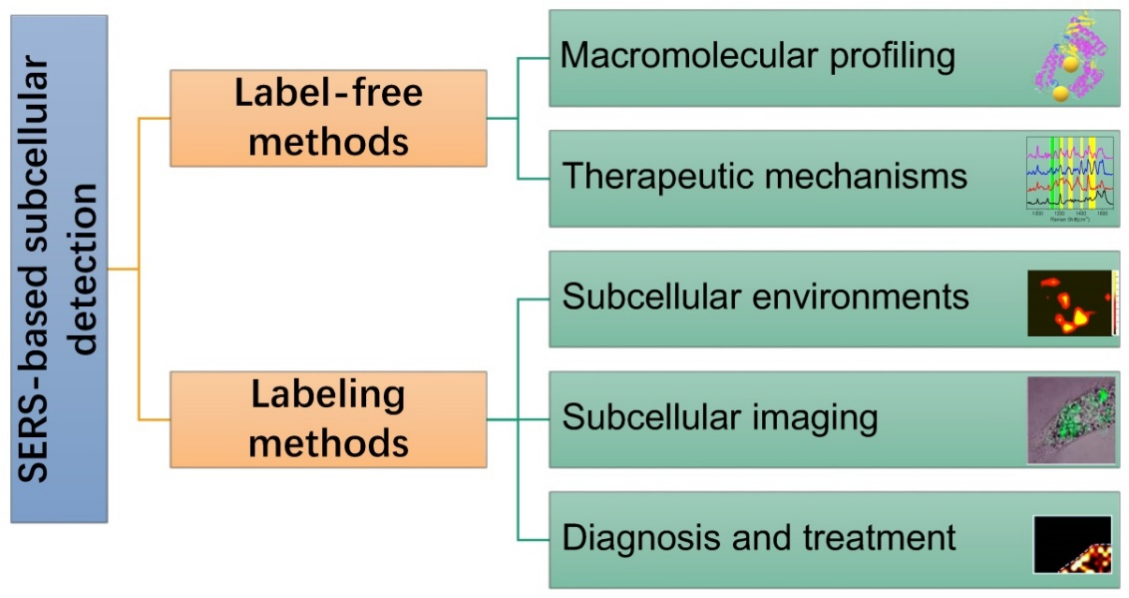
A scheme of the concepts/strategies of the SERS-based subcellular detections.

In this review, SERS as a useful analytical tool in subcellular components based detection and therapy were retrospected and summarized (Scheme 1). Two commonly used SERS strategies in biological applications, labeling and label-free methods, were briefly introduced. Some experimental notes were given, and SERS applications for the subcellular compartment detections, including cell membrane, nucleus, mitochondrion, and lysosome, were reviewed. The organelle-based therapy mechanism and the molecular dynamics during the therapeutic processes were presented as a frontier topic. We also described our concerns and perspectives on the future of subcellular SERS applications.

## Strategies for subcellular SERS studies

### Uniqueness and advantages of SERS for subcellular compartment analyses

SERS has gained ever-increasing attention, recently focusing on cellular studies. This high interest arises mainly from the uniqueness of SERS (molecular specificity and great sensitivity two-in-one) and highly matching between the SERS specialties and the demands of cellular/subcellular investigations. SERS exhibits applicability in subcellular detections from the following aspects.

**(1) SERS has an acceptable spatial resolution level for main cell compartments.** The cells and prominent organelles, such as cell nuclei (1-20 μm), mitochondria (0.5-1 μm), and lysosome (less than 1 μm), are all at the micrometer level 32, while the plasmonic nanometals are at the nanometer level. Thus, SERS can satisfy the spatial resolution requirements in subcellular detections and give good spatial discrimination on the subcellular domains *via* the commercial confocal Raman microspectrometers. SERS micro-imaging not only can provide two-dimensional information, but also can reveal surface, interface, or even layer-by-layer information of cells and organelles, enabling three-dimensional chemical structure reconstruction 33.

**(2) The localized field enhancement effect allows for selective measurements of aimed locations.** SERS is a highly surface-dependent effect 34. The physical mechanism discloses that the localized surface plasmon resonance effect provides an amplified electromagnetic field that only localizes around the metal surface. Moreover, the chemical mechanism of SERS that exists in typical charge transfer processes can't stand without the surface. Thus, only the probed molecules extremely close to the nanometal surfaces can be detected. It becomes possible to selectively analyze the interested analytes with the help of the organelle-targeted metal nanoparticles (MNPs) previously internalized in a specific organelle.

**(3) SERS can explore living cells in an* in-situ* way.** SERS features the common points of optical approaches that are non or less invasive and show minimum biological sample damage 33, 35. It can be applied for the living cell analysis and the dynamic tracking of physiological and metabolism processes.

### Label-free/labeling methods

**Scheme 2:**
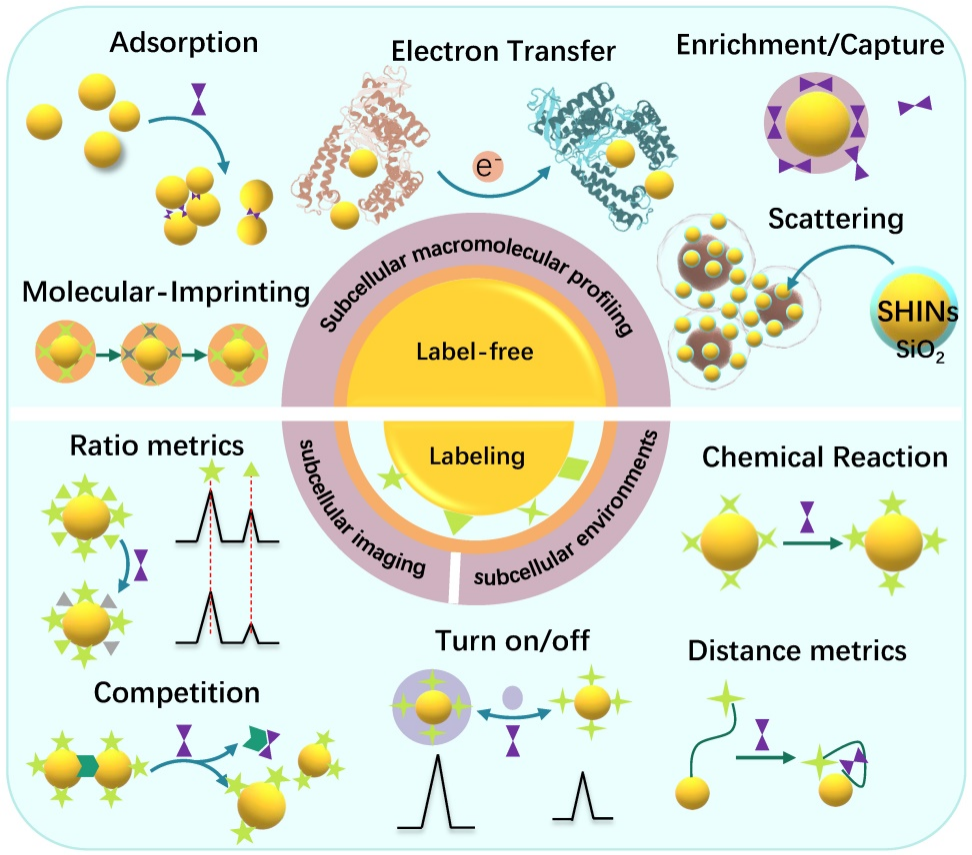
Illustration of the label-free and labeling detection methods.

Subcellular SERS studies follow the conventional SERS detection strategies, which can be sorted as direct/indirect measurements. In some descriptions, they are mentioned as label-free/labeling methods, as shown in Scheme 2.

The label-free method discloses the molecular vibration information of analytes by placing them near the SERS substrates, or the analytes directly contacting the SERS substrates 26. The most significant advantage of the label-free SERS is that it can provide the intrinsic information of the molecules and avoid false-positive. The direct SERS can analyze molecular dynamic changes in some life activities and the interactions between the analytes and other substances 36. Besides, the label-free method can provide the conformation and orientation information of adsorbed biomolecules. People have successfully applied the label-free SERS for the differentiation and identification of cells or bacteria 37, 38, cell activities such as cell mitosis 24, apoptosis 39, metabolism 40, intracellular biological macromolecules responding to external stimuli, and other metabolites in living cells 41.

Besides the label-free SERS, another SERS analytical strategy is the SERS labeling method. The SERS reporters and the recognition ligands are modified on the surface of plasmonic NPs, and these integrated complexes are called SERS tags or SERS labels 42, 43. In a stimuli-responsive process, when the target appears, the ligands can respond, causing the SERS signal changes of reporters 26. Alternatively, both SERS activity and recognition function are integrated into one, simplifying the labeling strategy 44. Either the target molecules or the physical or chemical properties of the samples can trigger the SERS signals of the reporter molecules to change. SERS labeling methods are suitable for the detection systems that the analyte without identifiable Raman signal or the target can't be detected by the label-free methods, especially some small molecules or subcellular microenvironments, e.g., local pH, and intracellular temperature changes and some molecules with low Raman activity.

The quantitative sensing mechanisms of SERS nanosensors are mainly cataloged in two types: (1) intensity (ratio)-dependent SERS nanosensors. By constructing or destroying the hotspot structures of the targets, or the signal changes relative to the internal standards, the “SERS on” or “SERS off” is entirely dependent on the concentration of analytes 45-47. (2) Raman shift changes of the SERS reporters. According to the interactions between the reporters with the analytes or the environments, e.g., charge transfer 48, chemical reaction 49, and host-guest interaction 50, the Raman band shifts of the SERS reporter will be observed, and the correlation between the peak shift and analyte concentration will be built.

SERS labeling methods exhibit broad usage in multi-component detection and imaging 51-53. The labeling methods lose the advantage of providing material intrinsic information relative to the label-free detections; however, they possess ultra-high detection sensitivity for quantitative determinations. In SERS imaging and mapping experiments, traceable SERS tags can provide high sensitivity comparable to many fluorescence dyes. Still, its narrow fingerprint bands allow the greater possibility for multiplex detections than the fluorescent dyes that are limited to only three channels (blue, green, and red) in the visible light range.

### Fundamentals of subcellular targeted detection and therapy

For subcellular targeting researches, delivering the SERS-active nanoplatforms into specific subcellular compartments is one of the most critical steps. For nanomaterials internalization, phagocytosis and pinocytosis are two major cellular internalization mechanisms for exogenous NPs. Most particles will arrive in the lysosome or cytosol eventually. To help the SERS-active nanoplatforms arriving in our interested organelles, so far, two main strategies have been employed and developed to deliver nanocarriers to specific subcellular organelles, noted as passive targeting and active targeting mechanisms.

#### Passive targeting

Passive targeting refers to the selective accumulation of NPs or nanosystems in specific organelles due to the physical, chemical, and pharmacological factors of the organelles (membrane permeability, potential, and internal environment, etc.) and the inherent properties of NPs, including size, shape, and surface charge. For example, small molecules such as water and some ions can freely enter and exit the nucleus since the nuclear pores on the surface of the nuclear membrane are about 9 nm in size 54. Accordingly, the nuclear-targeted delivery of nanomaterials can be successfully achieved by controlling their molecular weight or size 54. With the rapid development of nanotechnology, various ultra-small nanomaterials were developed as potential nuclear pore penetration materials 55. The internalization of nanomaterials in mitochondria is through a different way. Mitochondrion has a membrane potential of nearly -180 mV, and such a negative potential allows positively charged nanomaterials in the mitochondria autonomously 56. Accordingly, many mitochondrion-targeting synthetic materials were designed by enriching them with positive charges, e.g., a positively ratiometric Raman probe was prepared to achieve the selective mitochondria targeting and live-cell imaging of hydrogen sulfide in cancer cells 57. Although passive targeting shows apparent organelle-targeting to a certain degree, it is still limited in its applicability for all kinds of NPs. For example, only ultra-small nanomaterials can be delivered into the nucleus; however, for the NPs with larger sizes, it will be challenging to deliver them to the nucleus if no targeting ligand modification. Therefore, passive targeting is not a universal method to achieve nucleus targeting, and the applications are limited.

#### Active targeting

Developing universal targeting strategies that are independent of the inherent natures of NPs and organelles is in great demand. Active targeting by modifying targeting ligands such as aptamers and targeting peptide sequences on the surface of the nanomaterials has been extensively exploited, in which the specific targeting peptide sequences are the most commonly used targeting ligands. The surface decoration of the cell-penetrating peptide (RGD, RGDRGDRGDRGDPGC) is helpful for the internalization of nanomaterials. RGD is known to target αvβ6, an RGD receptor, as well as other αv integrins on the cell surface and assists the receptor-mediated endocytosis in cancer cells 58-60.

Shi's group reported a variety of nuclear targeting nano-delivery systems by conjugating the nucleus targeting peptide on the surface of the nanomaterials for cancer targeting therapy 61-63. They used nuclear targeting sequence is the TAT peptide (YGRKKRRQRRR). This sequence can translocate NPs into cell nuclei by binding the import receptors importin α and β (karyopherin) and subsequently targeting the nuclear pore complex of cancer cells through entering their nuclei 64-66.

In order to obtain the mitochondrion-targeting, the mitochondrion-targeting peptide (MLALLGWWWFFSRKKC) 67, 68 and the membrane potential targeting based on the mitochondriotropics molecules are commonly used to facilitate the selective accumulation in mitochondria, such as the TPP cation conjugated NPs, 69, 70.

At present, the modification of nanocarriers with intelligent response-type lipophilic amines is the most effective strategy for realizing the long-stay of nanocarriers in the lysosome and prohibiting the nanocarriers from escaping from lysosomes. Lysosomes give an acidic microenvironment, which makes amino groups protonated. Thus, NPs with the protonated amino groups easily penetrate the lysosomal membrane, resulting in abundant accumulation of NPs in lysosomes. For other organelles except lysosomes, the microenvironment is in a neutral condition, which can't trigger the protonation reactions, and thus the lysosome-selective targeting is realized. 4-(2-hydroxyethyl) morpholine and N, N-dimethylethylenediamine are two commonly used lysosomal targeting groups, and both of them have been used for the surface decoration of nanomaterials 71, 72.

### Less-invasive interventional operation

**Figure 1:**
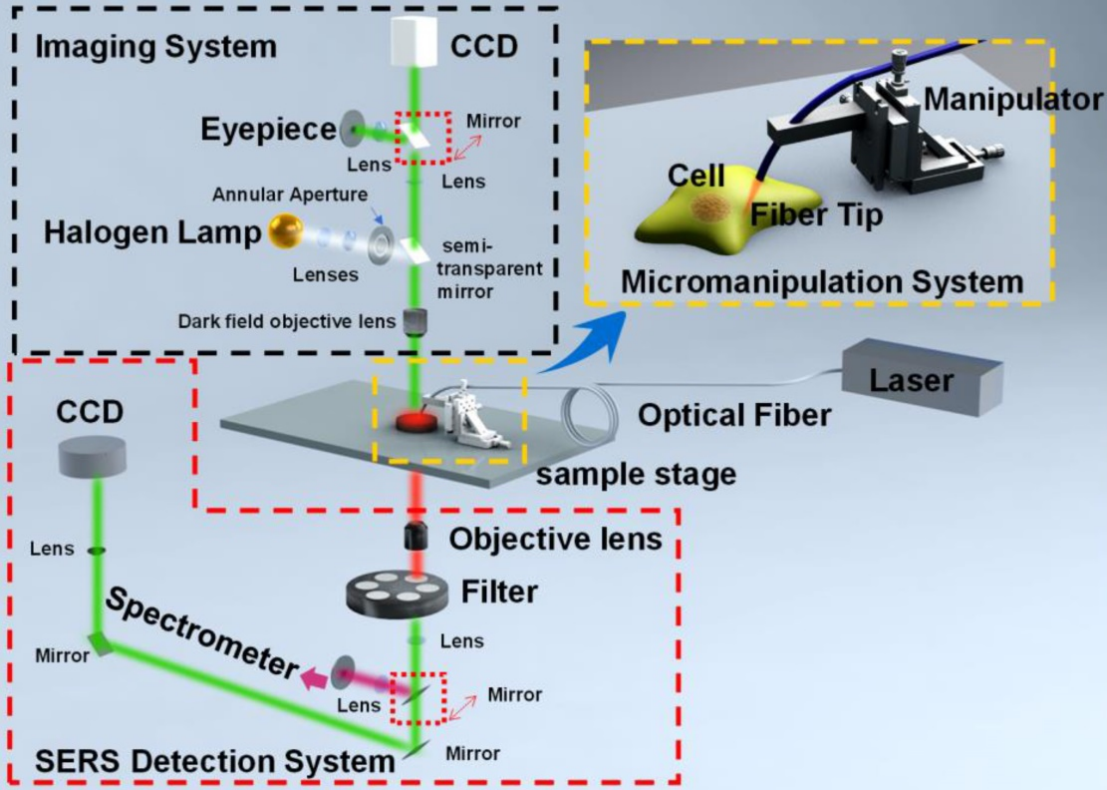
The schematic diagram of the micromanipulation-Raman detection system. Adapted with permission from 80 Copyright 2019 Elsevier B.V.

Optical fiber tip 73, 74, carbon nanotube 75, needle tip 76, 77, can be applied for micro-operation-based *in vivo* cell SERS measurements 79. These methods break through the traditional SERS substrates in detecting micro-regions by the initiative or passive internalization of MNPs. Differently, they can accurately reach the interesting positions of cells. The tips are mainly composed of single-core quartz fiber, hollow borosilicate glass tubes, or hollow quartz glass tubes, which can be easily fabricated into sharp tips and have good toughness during cell insertion. The ends of microprobes are functionalized with plasmonic NPs, which are highly sensitive to the intracellular microenvironments, enabling the SERS detections of characteristic spectral signatures of cell nucleus and cytoplasm. These nanotips were fixed on a microscope, while a micro-manipulation system is needed (Figure 1). The microscope with 10× objective was used to observe and guide the insertion of the nanotip under the white light. After the tip approaching the surface of cells, a high power objective was switched to record the SERS spectra of the cellular compartments with the laser irradiation and high-resolution spectrometer. By dynamic spectral processing and analysis, these microprobes provide an effective method for monitoring the dynamics and physiology of a single cell 40, 79, 80.

### Notes for experimental aspects

#### Impacts of SERS-active MNPs on cell physiological activity

Since these internalized MNPs are exogenous matters for cells, they will bring certain interference to the cells. Commonly, for the intracellular SERS measurements, the AuNPs are more acceptable than AgNPs for the consideration of better biocompatibility of Au, especially for the nanotherapeutic experiments in living bodies. So far, Au nanorods (AuNRs) 81, stars 82, spheres 36, and core/shell NPs 83, gap-enhanced Raman tags (GERTs) 84, 85, have been reported for intracellular compartment studies.

The concentration of the SERS-active nanoprobes should be assessed before intracellular SERS measurements, by testing the cellular viability after the cells co-culture with nanoprobes for different time (experimental time). The cell viability can be simply determined by using the MTT (3-(4,5-dimethylthiazol-2-yl)-2,5-diphenyltetrazolium bromide) or WST-1 (2-(4-iodophenyl)-3-(4-nitrophenyl)-5-(2, 4-disulfophenyl)-2H, tetrazolium monosodium salt) etc. assays, in which the cells were properly metabolized by a dye. They undergo visible color changes that are monitored by spectrophotometer, while the cells incapable of metabolizing the dye remain colorless. Also, flow cytometry is another powerful tool for cell viability tests. Taking spherical AuNPs as an example, over 85% of cellular viability based on the MTT assay is considered low/no toxicity and widely acceptable for intracellular experiments. Above 90% of cell viability is preferred. Otherwise, they will cause severe cell apoptosis. However, in order to achieve high SERS sensitivity, more SERS nanoprobes will be helpful. Thus, a trade-off concentration of nanoprobe is expected.

#### Surface engineering of MNPs

SERS tags are usually functionalized by both Raman reporters with distinguished signatures and the functional coating layers that endow the SERS tags with biocompatibility/stability and/or targeting bioconjugation. In many reported SERS nanoprobes, the surface coating of polyethylene glycol (PEG) is preferred, which offers protection from the chemical and physical environments, minimizing non-specific binding and prolonging blood circulation lifetimes in living body experiments 86. PEG is also a feasible linker for the conjugation of biomolecules (e.g., antibodies and peptides), and it can anchor biomolecules to noble metal surfaces through the carboxyl or amino groups. However, some ligands, e.g., cetyltrimethylammonium bromide (CTAB), may give identifiable peaks in the SERS profiles. These functional ligands may compete for the limited surface of nanoprobes with the probed components, which goes against the SERS detections of cellular compartments. So, the Raman signals of the used ligands should be confirmed in advance to make sure their contributions are distinguishable. In some cases, the bands of ligands can also be the internal standards for SERS intensity calibration in quantitative determinations.

The addition of functional coating molecules onto the NP surface tends to affect colloidal stability and brings irreversible NP aggregation, especially in the centrifugation step. This issue also limits the large-scale SERS tag synthesis. How to avoid the colloid aggregation during the SERS nanoprobe preparation is very skillful and bothered each beginner. Thus, the ligand amount must be strictly controlled, and the surface decoration of colloids needs well designed.

The surfaces of MNPs are active for the unspecific bindings of uninterested molecules. To avoid these SERS background signals, commonly used biocompatible protective layers - silica or bovine serum albumin (BSA) coatings 87 were utilized to block the nanoprobe surface and both of which can minimize the particle aggregations. Ren et al. proposed an iodide-modified AgNPs, and the iodide can avoid the nonspecific binding of molecules 88, remarkably improving the reliability and reproducibility of the label-free SERS detections.

#### Photostability and chemical stability of nanoprobes

Similar to the photobleaching of the fluorescent dyes used for imaging and bio-labeling, the SERS nanoprobes also face the fading problem. The irregular variation or fluctuation of SERS signals may cause the SERS blinking effect, which is the typical behavior of a single molecule caused by the diffusion effects of analytes (Brownian diffusion) 89 or the surface charge fluctuation-caused vibronic coupling 90. During long-term imaging, the intensity decay was also commonly observed, which is explained by the situations that the reporter molecules diffuse away from the hot spots or they are gradually destroyed by the local photothermal effect around hot spots. To maintain the SERS photostability of nanoprobes upon continuous laser irradiation, people synthesized the core-shell NPs with the embedded Raman reporters, which exhibit excellent photostability performance during the SERS imaging due to the metal protection shell layer and the off-resonance based low photothermal effect 91, 92.

A silica shell can also be a protective layer to maintain colloidal stability and prevent interaction with the external environments. The primary strategy for silica shell decoration is achieved by the mercaptotrimethoxysilane or aminopropyltrimethoxysilane (APTMS) and then reacted with sodium silicate or tetraethylorthosilicate. A remarkable example is the shell-isolated NPs (SHINs, Scheme 2). The chemical stability of the MNPs was significantly improved, and long-term storage was achieved 93.

#### Photodamage

The local photothermal energy not only burns the Raman reporters but also directly damages the cells and tissues during the SERS measurements. Though the photodamage effect of MNPs can be utilized for killing cancer cells according to their large absorption cross-sectional area of near-infrared light, in most SERS measurements of living cells or bodies, photodamage is undesirable. Laser power should be optimized for safety. Lim et al. 94 set different laser powers for the Raman imaging of human oral cancer cells (HSC-3), and the significant change of cell morphology and the cell death (red staining) was observed when 4.0 and 2.0 mW laser were set (785 nm wavelength Ti: Sapphire laser, 3900S, Spectra-Physics), while for 0.2 mW of laser power, no significant changes of cell morphology were observed. Xia et al. 95 quantified the photothermal treatment effect of 65 nm immune gold nanocages on SK-BR-3 breast cancer cells with a pulsed near-infrared laser (805 nm with a bandwidth of 54 nm, 4.77 W/cm^2^). In the absence of Au cages, the cells could alive under the laser power of 6.0 W/cm^2^ for 5 min, but they started to die at a laser power of 1.5 W/cm^2^ if the Au cages exist. In order to avoid the photodamage on the biosamples, a defocusing way can be applied to minimize the effects of laser damage if the vertical spatial resolution can be ignored 96.

## Subcellular compartment SERS detections

In this section, more detailed examples applying SERS label-free and labeling technologies for subcellular compartment detections will be provided, including subcellular macromolecular profiling, subcellular imaging, subcellular environments, cell membrane detections, and isolated organelles.

### Label-free SERS for subcellular macromolecular profiling

**Figure 2:**
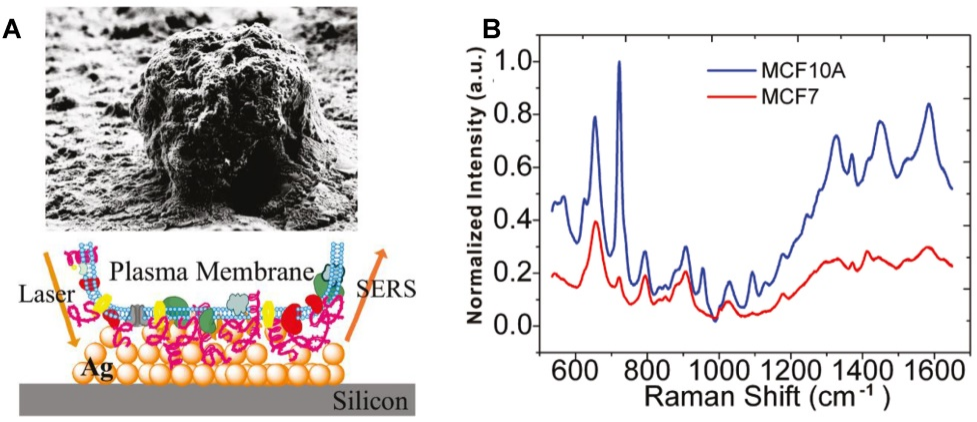
The SEM image of an individual cell and the field enhancement generated in the vicinity of AgNPs on the silicon. B SERS spectra of the MCF7 (red) and MCF10A (blue) breast cell lines obtained from the Ag decorated silicon. Adapted with permission from 97. Copyright 2010 American Chemical Society.

Researchers designed and synthesized various subcellular targeting nanoprobes to obtain the SERS spectra of different subcellular organelles by delivering noble metal nanomaterials (Au and Ag NPs) to the target locations. The surface of the cell membrane is rich in receptors and targets. In 2010, Yan et al. obtained the SERS spectra of the cellular surface of the tumor and nontumor cell lines under the assistance of the drop-coating AgNPs on a silicon wafer (Figure 2A). The quantitative analysis revealed that the characteristic differences between tumor and nontumor cells, especially in the spectral range from 600 to 900 cm^-1^ (Figure 2B), indicating the possibility of improving the precision of current cancer detection and staging approaches through the spectroscopic profiling of the cell surface cancer biomarkers 97.

Oyelere et al. prepared the AuNR-based nuclear targeting probes by the grafting of a simian virus (SV40) NLS peptide (CGGPKKKRKVGG) on the AuNRs through a thioalkyl-triazole linker 98. Based on these nanoprobes, the SERS spectra of malignant and normal epithelial cells were collected to reveal their differences in molecular structure information, which is expected to provide a new way for cancer cell detections. Xu et al. 99 developed a modified strategy in which 5-ethynyl-2'-deoxyuridine (Edu) was adopted as an internal label to locate the nuclear region accurately. In the case of the nuclear targeting AuNRs (decorated with NLS peptide), the accompanying signal of Edu in the Raman silent range can verify whether the SERS spectrum was recorded from the nucleus or not. Thus, the nanoprobe locating and *in situ* SERS detection of the cellular nucleus can be synchronously completed.

To obtain the SERS information of the entire mitochondria, Karatas et al. isolated mitochondria from A549 lung cancer cells and mixed them with AuNPs to obtain the SERS spectra of mitochondria 100. Since the redox state and conformation of cytochrome c in mitochondria can affect the overall state of the mitochondrial electron transport chain, as well as the lipid composition and the apoptosis-related processes, Brazhe et al. prepared a silver layered ring substrate to record the conformation and the redox state of the cytochrome c under various conditions 101.

**Figure 3:**
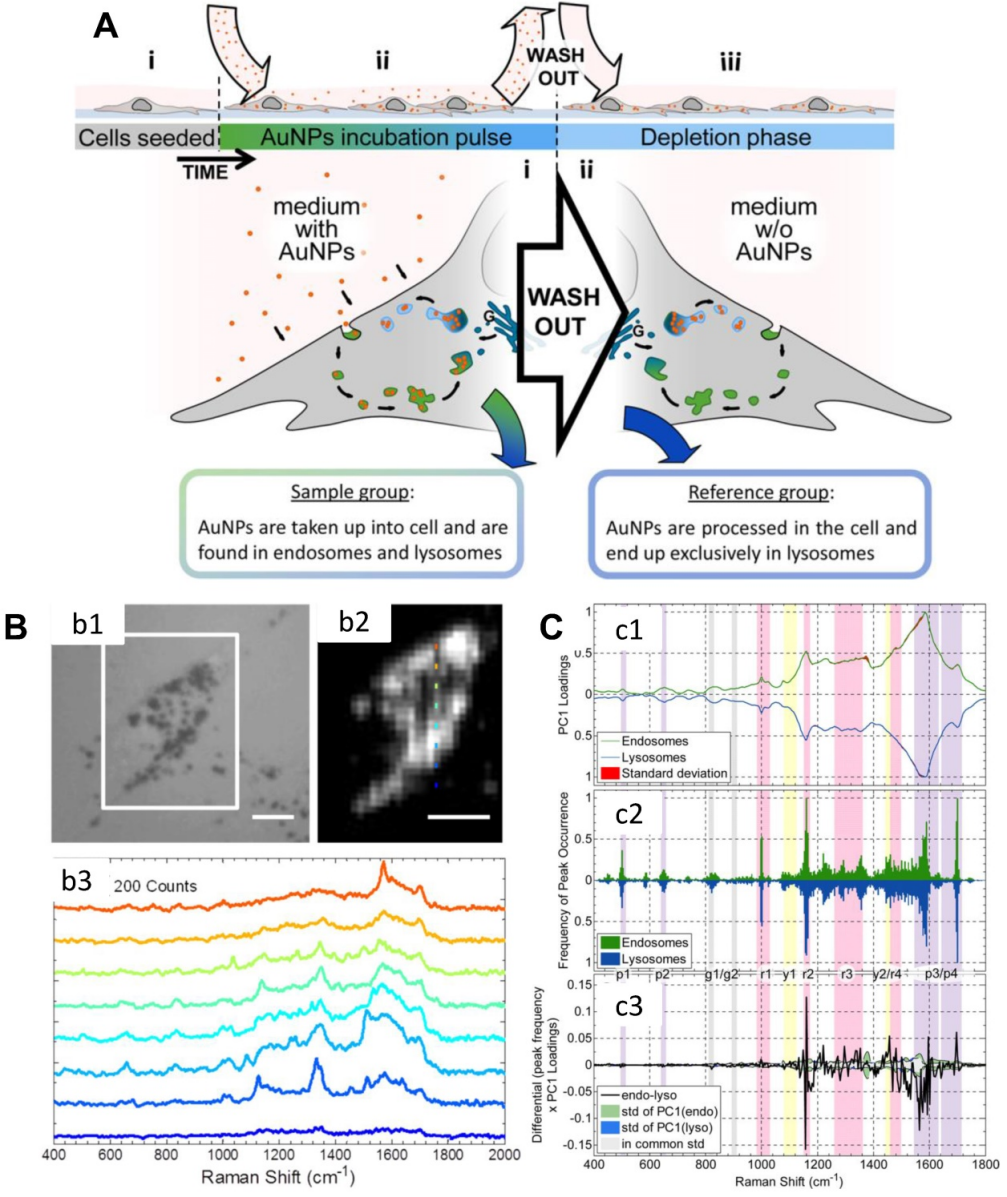
A) Scheme of the experimental design. B) (b1) The bright-field image of a cell; (b2) The corresponding SERS map of (b1) (integrated over 400-2000 cm^-1^); Raman spectra of the different positions of (b2) are shown in (b3). Scale bars: 10 µm. C) Analysis of spectra obtained from the cellular pathway. (c1) Normalized PC1 loadings of all extracted spectra originating from endosomes (green) and lysosomes (blue). (c2) The summary of the (normalized) occurrence probability of the SERS peaks of endosomes and lysosomes. (c3) Difference between the PC1 loadings (c1) according to the occurrence frequency of the peak (c2). The corresponding standard deviations of endosomes, lysosomes and both groups are shown in green, blue and gray. Adapted with permission from 103 Copyright 2015 American Chemical Society.

Nanomaterials enter cells through the clathrin-mediated endocytosis pathway, and they will accumulate in the lysosome or cytoplasm at the first stage 102. Mahajan et al. used label-free SERS to identify and visualize the stages of endocytosis of AuNPs in cells (Figure 3) 103. They cultured AuNPs with the cells by a pulse method to allow AuNPs to accumulate inside endosomes and lysosomes. Thus, they obtained the SERS signals of endosomes and lysosomes during the AuNPs' endocytosis. With the principal component analysis-linear discriminant analysis (PCA-LDA), several important life processes can be revealed by SERS 103.

**Figure 4:**
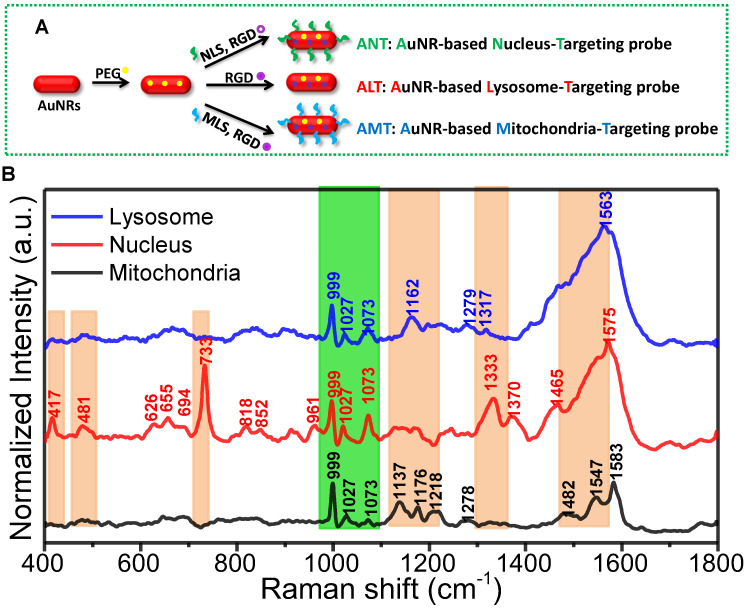
A) The preparation of the different organelle targeting nanoprobes. B) The normalized SERS spectra of the nucleus, mitochondria, and lysosome under the assistance of the subcellular targeted nanoprobes. The common and different spectra were shown in orange and green bands. Adapted with permission from 104. Copyright 2018 American Chemical Society.

To simultaneously observe and compare the molecular information of different organelles, Xu and Liang et al. designed and prepared the nuclear, mitochondrial and lysosomal targeting AuNR nanoprobes for recording the SERS spectra of three organelles (as shown in Figure 4A). First, the targeting ability of these nanoprobes was identified by super-resolution imaging and transmission electron microscopy (TEM). Under the assistance of these AuNRs-based nanoprobes, the macromolecular profiles of the three subcellular organelles were partly profiled and compared for the first time 104 (Figure 4B).

**Figure 5:**
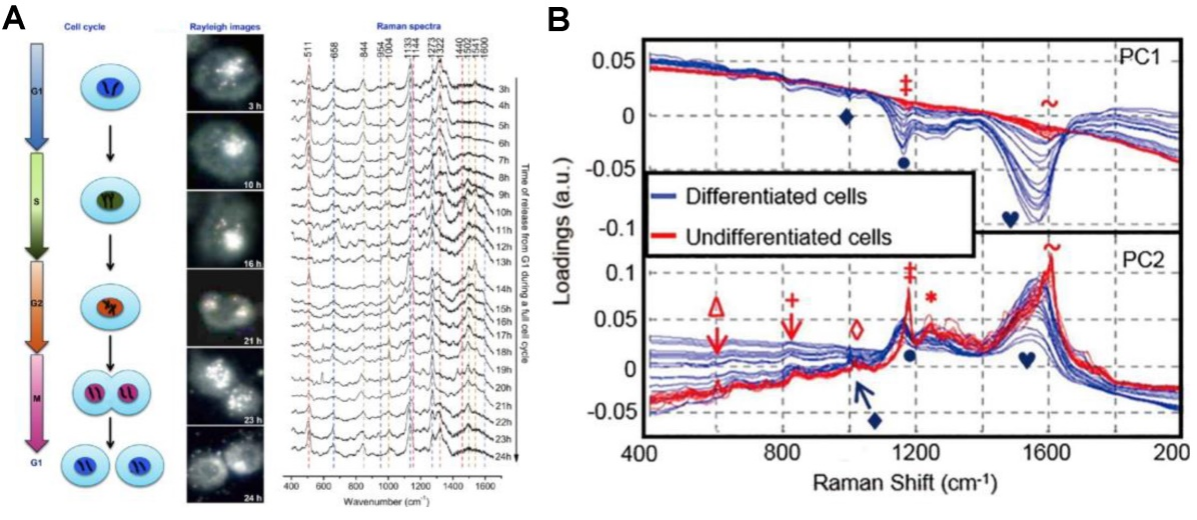
A) The process of a cell cycle, real-time dark-field images and the SERS spectra of the nucleus in a single HSC-3 cell during the complete cycle. B) PCA analysis of nuclear SERS spectra of undifferentiated and differentiated cells, shown as a scatter plot and 1D intensity plots for PC1 against PC2 scores. Adapted with permission from 24 and 105. Copyright 2012 and 2013 American Chemical Society.

In addition to the subcellular molecular profiling, the SERS strategy can also be used to record the subcellular spectra during dynamic cellular processes, including cell division, differentiation, and apoptosis. El-Sayed group demonstrated the real-time SERS monitoring of the molecular dynamics of mitosis in healthy and cancer cells at the molecular level (Figure 5A) 24. The nuclear targeting AuNPs were used to track the molecular changes of cancer and normal cells during the cell mitotic process. The results showed that the conformation rate of mitotic proteins from their α-helix structure to the β-sheet is higher in cancer cells during the mitosis process. However, in healthy cells, the existence of proteins is mainly in the α-helix conformation. The roles of protein conformation dynamics during mitosis in cancer development and many other diseases were revealed by this study. In 2013, Mahajan's group used the nuclear targeting probes to detect SERS spectra of different neuronal cells to obtain the SERS spectra of the cytoplasm and nucleus of neuronal cells (Figure 5B). SERS spectra were analyzed with the PCA to distinguish the differentiated and undifferentiated neuronal cells 105.

Although modifying the targeting peptide on the MNP surface can achieve effective organelle targeting, inevitably, some of them will randomly distribute in the cytoplasm, not in the targeted organelles, which limits the accuracy of detection in organelle based studies. The development of nano-tip technology effectively resolves this problem. In 2009, Vitol et al. developed the SERS-active nanoneedles for *in situ* molecular observation in cells for the first time 106. The prepared SERS-active nanoneedles can accurately locate in the nucleus and cytoplasm to accurately record the SERS spectra of the cytoplasm and nucleus. This approach provides a new path for cell injection, single-cell surgery, or *in situ* studies of organelle based cell processes.

The applications of label-free SERS in subcellular studies are mainly about the molecular information profiling of organelles such as proteins, DNA, lipids, and their dynamic changes in critical cellular processes 26, 35. In order to improve detection sensitivity, nonlinear spectroscopic techniques have been employed, e.g., stimulated Raman scattering (SRS) and coherent anti-Stokes Raman scattering (CARS) 107, 108, which are promising not only in cellular imaging but also in tissue imaging.

### SERS labels for subcellular imaging

**Figure 6:**
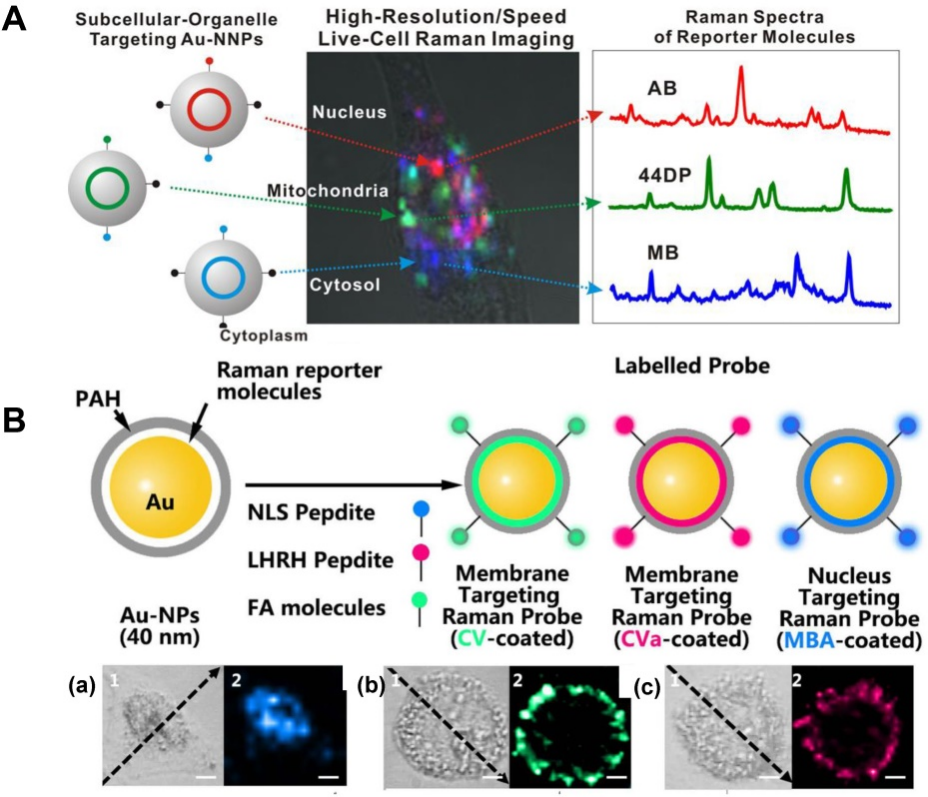
A Scheme of the Au@Au core-shell organelle targeting nanoprobes and the SERS spectra of the nucleus, mitochondria, and cytosol after the cell cultured with different organelle targeting nanoprobes. Adapted with permission from 94 Copyright 2015 American Chemical Society. B Scheme for the AuNPs coated with CV and CVa for targeting the membrane, and the MBA-coated AuNPs for targeting the nucleus (top). The corresponding bright-field and SERS imaging of a HeLa cell treated with the targeted nanoprobes of MBA-coated AuNPs (a), CV-coated AuNPs (b), and CVa-coated AuNPs (c) (bottom). Adapted with permission from 53 Copyright 2016 Macmillan Publishers Limited.

SERS tags with high sensitivity can be one type of location tracers at a subcellular level. In 2015, Lim et al. inserted three different Raman-active molecules into the gap of the Au@Au core-shell structure and further modified them with different organelle targeting peptides for organelle location and imaging 94. The hot spots generated by the nanogap allow them to create high-speed, high-resolution, multiplexed subcellular organelle imaging (Figure 6A).

The expression of the cell surface species, such as glycoproteins and glycolipids, can be the indexes of various diseases. *In situ* visualization of them on cell membranes may provide the correlations of proteins, lipids, and glycosylation with the developing states and the metastasis of diseases. Shen et al. prepared the nucleus and membrane targeting nanoprobes by modifying AuNPs with different targeting groups and reporters 53. The high-resolution 3D image of a single cell, especially the cell membrane and nucleus, were respectively obtained. The chemical properties of the nucleus and the distribution of folic acid (FA) and luteinizing hormone-releasing hormone (LHRH) on the surface of the cell membrane were accurately located and detected (Figure 6B). A zone-controllable SERS approach was developed by Ju et al. for SERS imaging the protein-specific glycosylation on the cell surface using two typed nanoprobes. The AuNPs (10 nm)-based nanoprobe was designed by the surface functionalization of Raman reporters and dibenzocyclooctyne-amine. This nanoprobe not only displays negligible SERS signals, but also can recognize and link the azide-tagged glycan on the cell membrane surface *via* the click reaction. The aptamer modified AuNPs (30 or 40 nm) that can specifically identify the target proteins were then used for amplifying SERS signal by forming nanoaggregates. This method was used for *in situ* imaging of sialic acid (SA) on the target protein epithelial cell adhesion molecule (EpCAM), as well as their dynamic changes during drug treatment 109. Subsequently, Ju's group developed another nanoprobe by decorating the Au nanoflowers with the benzoic group functionalization, which were used as a bridge for both recognition of the target SA and assembly of poly(nacetylneuraminic acid) modified AuNPs. *Via* the plasmonic coupling of these two kinds of nanoprobes in a single core-multi satellite nanostructure, they realized the sensitive SERS imaging of SA on the surface of living cells. Moreover, they also designed a micro-competition system for the simultaneous quantification of multiple glycans on cell surfaces with SERS 110.

Based on the recognition of the benzoic group with SA, Xu et al. developed a 4-mercaptophenylboronic acid (MPBA)-functionalized AgNP-based SERS nanosensor to *in situ* study sialoglycan levels in different cell types and the dynamic expression processes of them under the physiological conditions 44. This nanosensor is unique and multifunctional due to its three-in-one role involving the Raman signal enhancer (AgNPs), the sensing reporter of MPBA, and the target receptor. When it binds to sialoglycans, the vibrational modes of MPBA will change, which can be used to track the sialoglycan expression on cell membranes. Recently, in combination with the microfluidic droplet system, they established a plasmonic microdroplet platform for the SA expression in a single cell *via* the MPBA@AgNPs SERS nanosensor 111, which avoids the interference of the neighbor cells. The single-cell SA expressions on different cell lines (HepG2, SGC-7901, MCF-7, and BNL.CL2) were compared 111. The single-cell analysis of the sialoglycan expression reveals the cellular diversity and heterogeneity.

### SERS nanosensors for subcellular environments

Subcellular microenvironments such as pH, the content of ROS, biomolecules, and redox potential are important parameters for ensuring normal cell growth, proliferation, and others 112, 113. However, any fluctuation of subcellular microenvironments can result in various disorders. Therefore, the *in situ* detections of the subcellular microenvironments will facilitate the understanding of the mechanisms of subcellular related diseases during the occurrence and development processes. In this section, we will introduce some recent progress in SERS tag-based subcellular microenvironment sensing, especially intracellular pH detections.

In 2020, Choi et al. reported an efficient SERS-based immunoassay technique for protein quantification and imaging. Their method can reliably estimate the protein level at a sub-picomolar detection limit 114. Besides, Ye et al. designed the SERS core-shell NPs with an embedded standard for the non-destructive detection of cholesterol 115. Wang et al. developed a simple SERS-based nanotip system to perform subcellular localization and detect nucleolin (NCL) in a single living cell based on the aptamer recognition 116. Their detection platform was available for multiplex targets by decorating the tips with different aptamers. Similarly, in combination with SERS technology and nanotip technology, Xu et al. designed an optical waveguide excited pH SERS-active system by modifying a pH-responsive molecule, 4-mercaptopyridine (Mpy), on the optical fiber tip 80. The system can detect the pH of the nucleus and cytoplasm. The results showed that the nucleus had a lower pH value than the cytoplasm. Moreover, the probe was used to evaluate the pH environment of normal cells and cancer cells inside/outside the cell. In short, these developed platforms realize the real-time monitoring of the distribution and the content of biomolecules at the subcellular level.

**Figure 7:**
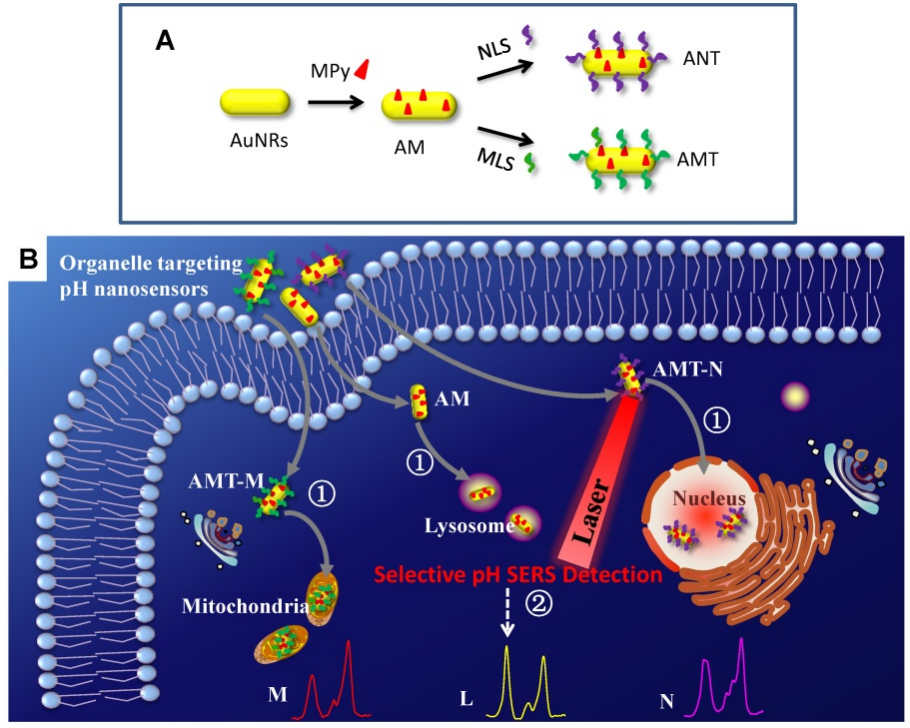
The preparation of organelle targeting pH nanosensors (A) and the procedure for subcellular pH determinations by SERS spectroscopy with the organelle targeting pH nanosensors (B). Adapted with permission from 117. Copyright 2018 Royal Society of Chemistry.

A series of SERS nanosensors with high sensitivity and organelle-targeting ability have been developed for monitoring the subcellular pHs 117. As shown in Figure 7, three nanosensors were obtained (Figure 7A) by modifying a pH-responsive molecule Mpy and different organelle targeting peptides on the surface of AuNRs. By monitoring the vibrational spectral changes of Mpy, the nuclear, mitochondrial, and lysosomal pHs were obtained and compared (Figure 7B). The developed SERS sensors can also explore more crucial physiological and biological processes related to subcellular pH.

In addition to those nanoprobes that should be internalized in cells for subsequent subcellular environment analyses, many needle-like optrodes and nanopipettes were utilized for tracing the single-cell low-copy proteins and components. Wang et al. decorated the outer surface of glass nanotubes with Au and Raman-active molecules to detect cancer biomarker, free iron ions and hemoglobin in a single cell *via* the aptamer hybridization 118. Liu et al. 119 modified monoclonal antibody or molecularly imprinted polymer on the surface of Au-coated nanotubes to achieve specific minimally invasive extraction and *in vitro* labeling detections. Gogotsi et al. 75 developed a carbon nanotube-tipped endoscope for intracellular biomolecular detection and realized the *in-situ* determination of the molecular composition of the organelles. Compared with the intracellular SERS applied by nanoprobes, the nanotip-based method can arrive at interesting locations. However, since the micromanipulation under the microscope has limited imaging resolution and low contrast for other organelles except for the cell nucleus, these optrodes are only applicable for detecting cell nucleus, cytoplasm, and extracellular regions.

### SERS measurement of isolated organelles

Although the rich and dynamic biomacromolecular information can be obtained by *in situ* SERS, there is still a problem unsolved on the difficulty to assign these bands because the intracellular biological environment is incredibly complicated, and there are some interference signals from other biomolecules. Therefore, some *ex-situ* strategy has emerged for structural analysis and quantitative analysis of the isolated organelles.

Culha et al. investigated the feasibility of SERS to understand the mode of interaction of AuNPs with the extracted mitochondria. They found that the AuNPs did not cause significant damage to mitochondria 100. Besides, Xu's group investigated the molecular information of mitochondria from cancer cells *in situ* with the assistant of mitochondrion-targeting nanoprobes, compared with the isolation-based *ex situ* method 120. In the following study, Xu et al. investigated the dynamic molecular changes of the extracted living mitochondria upon phototherapy by SERS, to elucidate the underlying molecular mechanisms associated with cell death induced by subcellular dysfunction. The result demonstrated that during the cell death process caused by phototherapy, the content of Phe in mitochondria started to increase. The cell apoptosis in this process was mainly due to the changes in DNA structures 121. Brazhe et al. probed cytochrome c and monitored the redox state and conformation of cytochrome c in the electron transport chain in living isolated mitochondria by SERS 101. They further studied living cells and the isolated mitochondria by SERS, and they achieved the redox state and the conformation of cytochromes in a natural cell environment 122. The accurate molecular information of the isolated mitochondria is essential for profoundly understanding the structure and function of mitochondria in cellular biological processes.

Nucleus is a place of genetic material storage and possesses a high-density place in one cell. Cytobiological methods for cell nucleus related studies start with the extraction processes of intranuclear components with many cell lysis buffers, following by the structural characterizations and quantitative analysis of the extracted components. The* in situ* SERS strategy to selectively measure intracellular biomolecules was compared with the *ex-situ* SERS strategy 123. This study described the accurate SERS profiles of the pure cell nuclei, the whole nuclear proteins/DNAs, and the DNA/protein extraction reagents as well 123. This is the first step in establishing a Ramanomics database of nuclear molecules.

## Therapeutic mechanisms and effects at subcellular compartment level revealed by SERS

At present, with a deeper understanding of the structure and function of organelles, the designs of the organelle targeting platforms based on chemotherapy, photothermal therapy (PTT), photodynamic therapy (PDT), and other multi-mode therapeutic platforms have attracted much attention. The most significant advantage of SERS technology in this field is that it can realize the real-time monitor of the biomolecular changes and the quantitative assay of small molecules in living cells.

### Subcellular compartment information revealed by SERS during therapies

It is unique to look at the process and mechanism of disease treatments from the organelles' Ramanomics. In 2013, the El-Sayed group developed a nuclear targeting plasmon-enhanced single-cell imaging technology to study the molecular changes of the nucleus, before and after the cells were treated with anticancer drugs 39. They further used the nuclear-targeted AuNPs as the PTT reagents to study the biomolecular changes in the nucleus during PTT 124. The results proved that during the PTT process, the molecular structures of proteins and lipids in the nuclei would change, causing cell death. The molecular structure changes have nothing to do with the size of nanomaterials and the intensity of light. This study provides us useful information to understand the mechanism of PTT induced cell death at the molecular level, which is vital for understanding the therapeutic mechanism of nuclear-targeted PTT and efficacy optimization. A further study based on mass spectrometry combined metabolomics and proteomics with SERS spectroscopy to discuss the PTT process 125. They also studied the changes of nuclear molecules under the action of ultraviolet light 41, high oxygen content 126, and AgNPs 127. These advances paved a new way for the SERS analyses of therapeutic mechanisms from the subcellular level.

Moreover, the therapeutic plasmonic SERS nanoprobes with organelle-targeting features were designed to study the different responses of cancer cells and normal cells upon to PTT. The nanoprobe composed of porous Ag/Au nanoshells and carbon dots, was prepared for the PTT of cells (Hela, L929, and H8 cells) at organelle level (nucleus and mitochondrion) to assess the heat tolerance mechanism by *in situ* SERS 128. The contents of tryptophan, phenylalanine (Phe), and tyrosine in HeLa cells were found more evidently increased than those of L929 and H8 cells during the PPT-induced cell death process. From the mitochondrial point of view, the PPT-induced cell apoptosis for HeLa cells mainly stems from (or is regulated through) cellular thermal stress-responsive proteins, while the damages of L929 and H8 cells seem more related to DNA. Different molecular stress responses at the single-cell level will help us understand the underlying mechanisms of the cell death induced by PTT.

In addition to PTT, changes in the SERS spectrum of the nucleus during PDT were also investigated. The B16 cell line (a murine melanoma cell line) was chosen. The results evidenced the protein degradation and the DNA fragmentation in the therapeutic process, which is meaningful not only for exploring the PDT molecular mechanisms but also for the assessment of the dosage of photosensitive reagents 129.

Besides phototherapies, electrical stimulation is another clinically permitted therapeutic mode. Its curative effect was also revealed by subcellular SERS, reported by Jin et al. 130. The different responses of the nuclei and mitochondria between normal and cancer cells under electrical stimulation were investigated by the nucleus- and mitochondrion-targeting AuNPs. The results disclosed a common electrosensory and self-repair biofunction of cells and its related Phe metabolism response.

**Figure 8:**
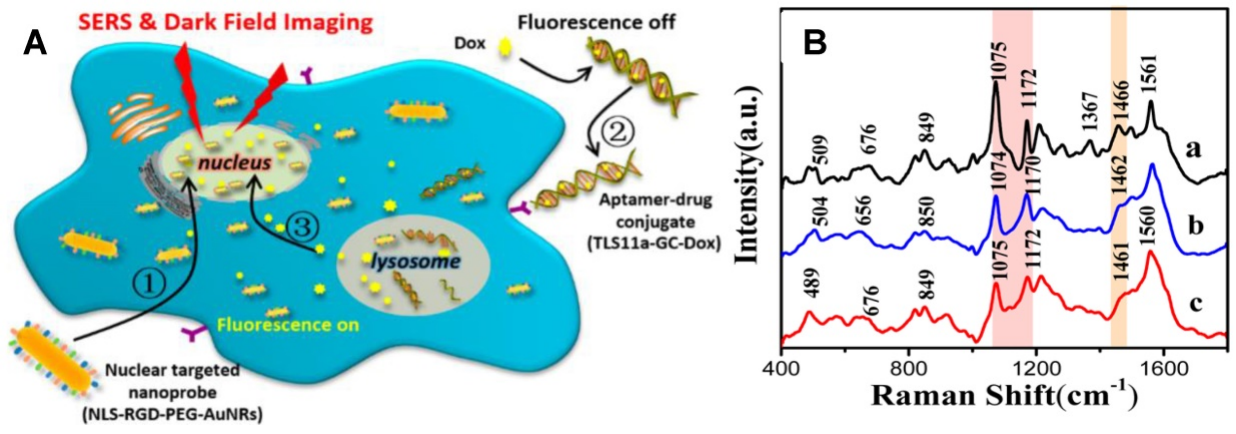
A The schematic illustration of the exploration of the therapeutic effect of aptamer/drug conjugates acting on HepG2 cells by SERS and dark-field imaging with the of nuclear targeting nanoprobes. B SERS spectra of HepG2 cells treated with nuclear-targeted nanoprobes and (a) and further with 4 µM TLS11a-GC-Dox conjugates (b) or 15 µM free Dox (c) for 24 h, respectively. Adapted with permission from 131 Copyright 2017 American Chemical Society.

Chemotherapy is an important way for tumor treatments. Xu et al. used AuNRs-based nuclear targeting probes to study the effects of DNA targeting molecules (Hoechst33342) and anticancer drugs (doxorubicin, Dox) on the nucleus 81. Spectral analysis showed that both molecules acting on the nucleus could change the spectral information of the nucleus. The DNA and protein structures in the nucleus were both influenced; however, the molecular mechanisms of the two molecules acting on the nucleus were different. Hoechst33342 executes its role mainly through acting on the DNA bases to affect vibrational modes of DNA and proteins. However, Dox principally induces DNA damage to achieve anticancer effects. Subsequently, the treatment process of a targeting aptamer/Dox (TLS11a-GC-Dox) drug delivery system on the nucleus was explored by SERS and dark-field technology (Figure 8) 131. The cell morphology shrunk severely during the treatment, and the DNA and protein structures in the nuclei also changed accordingly. This is the first report to gain insight into the treatment mechanism of a targeted drug by SERS.

### Subcellular microenvironments revealed by SERS during therapy

In addition to directly detecting the changes of biomolecules in organelles, detecting the intracellular microenvironment during treatments can help us better understand the therapeutic mechanism and the important role of organelles in the therapeutic processes. In 2016, Yang's group used the AuNRs with the decoration of pH-responsive molecules and a layer of the peptide as a protective layer to carry out the SERS detection for lysosomal pH during PTT 132. The intramitochondrial pH evolutions during the PDT process were also revealed by Xu's study, in which the Mpy-coated AuNRs with the mitochondrion-targeting peptide decoration were cultured with three kinds of cells before the PDT 133. When cells were irradiated with a LED for 1 min to trigger the photosensitive reagents releasing ROS, the mitochondrial pH significantly decreased, causing the early stage of apoptosis. With the continuous extension of the illumination time, the pH value in mitochondria increased, suggesting that the cells had self-defense and self-regulation function. The ROS SERS assay suggested that the LO2 cells have better resistance to ROS than two kinds of cancer cells (HepG2 and MCF-7).

Besides the pH environment, ROS changes are also crucial for the understanding of the mechanism of PTT and PDT. Recently, an interference-free SERS-active nanoprobe for intracellular ROS detection was developed by Xu et al 134. This nanoprobe was composed of an Au core-Ag shell (Au@Ag NPs) with a SERS reporter (4-mercaptobenzonitrile) resided in the gap of the core-shell. Intracellular ROS can etch the Ag shell and dramatically decreases the SERS intensity of the SERS reporters. The Raman band of the reporter used in this study is located in the cellular Raman-silent region (1800-2800 cm^-1^), which eliminates interference possibility from cellular molecules. By using these Au@Ag NPs, the ROS dynamic changes at mitochondria during the PTT process could be revealed by the Raman mapping. Results show that after irradiation by an 808 nm laser, the SERS intensities of the peak at 2226 cm^-1^ gradually decreased due to the ROS production during the PTT process. This study also demonstrated that during the PTT process, the ROS-induced cell death is a major reason in comparison to the heat-induced cell death.

### Integration of SERS diagnosis and treatment

As a powerful spectroscopic tool, SERS has been employed to detect the organelles and components at the subcellular level, and reveal the action mechanisms of some therapeutic means through the molecular fingerprint information. By integrating the diagnosis and therapy, SERS nanotags could track the release of the drugs in cells and realize tumor treatment at the same time.

In 2016, Cui et al. fabricated a multifunctional nanocarrier for tracking the intracellular Dox release process based on the acidic environment in lysosomes, achieving the killing effect on cancer cells. Furthermore, the detailed release process and the carrier distribution were recorded by SERS *via* the environmental pH changes during cell endocytosis 135. In the same way, Chen et al. investigated the intracellular SERS performance of the Dox loaded Ag-mesoporous silica nanocarriers, and the results demonstrated that the nanocarriers could preserve the SERS signal to realize the targeted detection and traceable drug delivery, which was favorable for easy endocytosis, pH-responsive drug release and simultaneous SERS imaging. This work highlighted the potential of Janus Ag-MSN nanocarriers as a practical tool for efficient cancer therapy 136.

In addition to the implementation of tracking drug delivery by SERS, some cancer cell treatment strategies with SERS tags were also developed to support therapy or feedback therapeutic efficacy. For example, Wang et al. prepared a novel traceable and targeted drug delivery nanosystem based on Au nanoflowers (AuNFs). This nanosystem has a high drug encapsulation capability and an intra-lysosome pH-controlled release feature. The AuNF-based nanocarriers were applied for efficient intracellular SERS imaging-guided chemo-phototherapy, showing great potential in facilitating the accurate* in vivo* treatment of tumors 137. In a recent study, Jin and Xu et al. developed an intelligent biodegradable nanoreactor based on the tumor microenvironmental response for the chemo-starvation synergistic treatment of tumors. The nanoreactor was composed of an assembly of the 4-mercaptobenzonitrile-decorated AgNPs (AgNPs@MBN) of the glucose oxidase (GOx)-loaded metal-organic-framework (ZIF-8@GOx). The ZIF-8@GOx part has an intelligent response to the acidic microenvironment of the cancerous lysosomes, enabling the release of drugs (GOx) to trigger the catalytic cascade reaction in the tumor. The surface element (AgNPs@MBN) of the nanoreactor has the function of *in-situ* SERS reporting glucose level in cells based on a ''turn-off'' mechanism (intensity decrease) of the Raman reporter (MBN) induced by the H_2_O_2_ etching effect on the AgNPs. Thus, the enzymatic activity of GOx and the degradability of the nanoreactor in an acidic environment can be monitored; that is, this nanosensor can immediately feedback the curative effect by SERS 138.

## Conclusions and future directions

This review summarized recent progress in subcellular compartment studies using SERS as a powerful analytical tool. The applications of the SERS-based detection schemes in subcellular structures studies were reviewed (Table 1), including the direct subcellular analysis, investigation of biological processes, as well as SERS-based indirect analysis including subcellular imaging and the SERS sensing of the cellular environment, including pH, ROS, and other vital components. Moreover, the organelle-targeting therapeutic mechanisms including the anticancer drugs, PTT, PDT, and electric stimulation. The integration of SERS diagnosis and treatment were also discussed. These might be a reference for researchers interested in the intracellular SERS studies.

Looking back on the above publications, people have made progress in many aspects; however, many efforts should be exerted no matter on the bio-SERS experimental aspects or on the extensive SERS applications in the subcellular studies, because this subject is full of challenges and many technical problems still exist. Many new technologies are involved in Raman analytical methods, and their integration will bring a brighter future for intracellular SERS studies. In the following, we listed six unsolved problems in the intracellular SERS experiments. According to these problems, a perspective for future studies is given. And some of them are already on the way to reality.

### Automation of synthesis of SERS nanoprobes

SERS tags and nanoprobes are all plasmonic MNP-based. So, to prepare SERS nanoprobes, we firstly synthesize metallic Au or Ag NPs. The shape and size are different in trials, which is one of the trouble issues leading to the poor repeatability and low accuracy of the SERS results. A robust method to synthesize monodisperse MNPs with high reproducibility and stability is in great demand. Except for many parameters, such as reagents, temperature, pH, concentration, charging sequence and mixing time, etc., the synthesis method is also essential. The seed-growth method 139 is an applicable and controllable method for the NP preparation due to its better size control. We can envision that automatic production lines and more rigorous quality control management are expected, which will make precise manufacturing of high-quality MNPs possible in the near future.

### The targeting ability of nanoplatforms in living cells

The SERS-based platform has been successfully used in the diagnosis and treatment of cancer *in vivo* due to SERS tags showing excellent signal intensity. Some organelle targeted-based drug release methods are also designed based on this point 140. However, the living cell has a complicated and crowded intracellular environment, and its physiological metabolism keeps going all the time. All of these bring uncertainty for the targeting ability of MNPs. Thus, the location of the SERS-based nanoplatforms should be carefully confirmed before SERS measurements and organelle-specific therapies. The optical fiber-based SERS probes and nanotip with the micromanipulators can accurately arrive in the interesting locations while tiny damage on the living cell membrane will occur, which might be a desirable solution for targeting the sites relative to the targeting MNPs for subcellular analysis. However, even a fine tip has a large apex angle, which will be an obstacle when it is applied to many indistinguishable organelles. Atomic force microscopy-based tip-enhanced Raman spectroscopy probe permits cell penetration 141, 142. Though the tip is very smaller, the shortcoming of this method is that the tip only can probe the location from top to down randomly. Thus, this topic is still open to welcome the integration of new techniques.

Because the targeting ability of the nanoprobes is one of the key issues for ensuring the reproducibility and accuracy of intracellular SERS, more specific targeting ligands and techniques are required to reduce the off-target effect of the nanoprobes. This progress often depends on the discoveries in bionics and the imitation of physiological processes such as viruses and bacteria. Recently, many precise diagnosis and medical methods, for example, gene-editing technique, click chemistry approach and fluorescence *in situ* hybridization methods, have been successfully applied for illuminating genome-scale and transcriptional activity in a single cell by fluorescent imaging 143. We believe many examples might be presented in combining these new techniques with subcellular SERS studies in the near future.

### Needs for more Raman reporters responding to small intracellular molecules

There are still too few molecules available for intracellular detection. Compared with fluorescent sensing molecules, the choice of responsive Raman reporting molecules is very limited, especially in the detection of small molecules in cells. Also, it is difficult for small molecules to trigger an effective signal amplification mechanism. So, it is still challenging to sense small intracellular molecules, as well as cellular metabolites. More efforts are required to involve in in the synthesis of sensing probe molecules and Raman reporters.

Since the limited surface area of a SERS-active NP, except giving the signal reporter with a larger Raman scattering cross-section and preferably having a band in bio-window range, the Raman reporters are expected to endow multi roles, for example, the bridging linker for identifying elements, the hydrophilic/hydrophobic regulator, the surface blocker and protector, and the modular for the plasmon feature of NPs, etc. The binding ways to metal are not limited to thoil, amino, and carboxyl groups anymore. In a recent study, Li and Tang et al. 144 developed the selenol-based reporter molecules, and found that the Au-Se interface is more stable than the biosensors constructed by the Au-S bond, displaying good resistance to abundant thiol under the biological condition and show better stability. The increasingly demanding molecular designs based on full consideration of all these issues is an irresistible trend, and many organic and pharmaceutical chemists might engage in developing high-quality and multifunctional Raman reporters.

### High-speed and ultra-high-resolution SERS imaging

Due to the Raman scattering cross-section of the molecules is about 10-30 cm^2^/sr, Raman-based subcellular imaging requires a relatively long exposure or integration time, making it difficult to image a subcellular compartment whose cellular changes can occur on the scale of milliseconds. The current SERS imaging speed seriously limits the detection in a high temporal resolution. Some promising improvements were achieved by using highly bright SERS nanotags. In 2019, Ye et al. 145, 146 reported GERTs with strong electromagnetic hot spots from interior subnanometer gaps, and these GERTs reached a Raman enhancement factor beyond 5×10^9^, the detection sensitivity down to a single-NP level, high-resolution cell imaging within 6 s and high-contrast. These nanoprobes may open new opportunities for rapid ultrasensitive biosensing and bioimaging. In addition to the highly bright SERS nanotags, Brolo's group 147 adopted the Airyscan photocell detector—a system composed of an array of 32 photomultiplier elements organized in a hexagonal tiling arrangement, and they demonstrated a single-molecule SERS imaging technique that could probe hotspot dynamics in an individual NP at 800,000 frames per second, gathering tens of millions of SERS data points, with ~7 nm spatial resolution 147. These results provide the SERS community with a high-speed experimental approach, indicating that rapid SERS imaging is achievable if neglecting ultra-high spectral resolution. Therefore, each advance of high-speed imaging technology will illuminate the SERS imaging field, which requires unremitting efforts on more delicate optical designs and devices.

Besides the imaging speed, spatial resolution is always pursued by researchers. Supra-resolution fluorescence imaging technology has become influential in visualizing the organelles and their interactions. Inspiringly, supra-resolution SERS imaging is already on the way. Using a high-density, uniform hot spot substrate (hot spot density 10^11^/cm^2^, 20 to 35 nm gap), Dana et al. 148 developed a SERS-stochastic optical reconstruction (STORM) technique that exhibits a spatially uniform distribution of spot locations with no identifiable sharp features to obtain the 20 nm resolution super-resolved SERS images, and they applied this technique for several complex biological architectures, e.g., the supramolecular self-assembled peptide networks, microalgae membranes, and eukaryotic cells. Lindquist's group 149 combined SERS direct detection with STORM by a 700 nm hexagonal periodic metal nanopore SERS hotspot array with a unit diameter of 150 nm to match a 660 nm laser, which causes the hotspot scintillation locating at 10 nm regions of the substrate to achieve the SERS-STORM imaging. They further employed an ultrathin silver island film to identify the molecular structures of the cell walls of two types of bacteria (Gram-negative bacteria and Gram-positive bacteria) 150. So far, there are no reports on SERS supra-resolution imaging to visualize the distributions of intracellular compartments with the help of SERS tags. SERS and Raman micro-imaging have been widely reported 151. Therefore, we believe that the rapid development of the supra-resolution imaging technology will one day be combined with SERS imaging technology, which will become a powerful tool for studying cell structure, morphology, mass transfer and so on.

### Establishment of an extensive and accurate subcellular biomolecule database for spectral assignments

Establishing a consolidated SERS database is imperative toward the practical application of SERS. Most subcellular SERS studies are currently based on the workgroup-specific database obtained under different technical and biological parameters or the experience summed up by the predecessors for cellular analysis. This hinders validation in large-scale studies and implementation in clinics. It can't work for the information of unknown samples, or selectively identifying molecular changes resulting from cellular processes in large and multidimensional data sets. Following SERS methodology standardization for subcellular studies, the most critical step is to establish a consolidated subcellular biomolecules SERS database. This database will provide the comprehensive spectral profiles of organelles, including the data of subcellular structures in different cells and the same subcellular compartment at different physiological states, for instance, in response to various stresses. It will be a reliable tool for the identification, discrimination of subcellular compartments, in-depth deciphering of mechanism, and even prediction.

### Combination of SERS and chemometric/intelligence algorithms

Although the sensitivity of Raman spectroscopy is sufficiently high to identify significant cellular macromolecular information and their variations existing between different cellular samples and different cellular processes *via* label-free SERS strategy, reasonable analysis and processing of the obtained data is still a challenge, no matter in the cellular level or in the sub-cellular level. Therefore, combined with advanced algorithms for demultiplexing or decoding the spectra of components will be promising in the near future, e.g., PCA-LDA 103. The application of chemometric methods in SERS spectroscopy will help us determine the changes between spectra and biomarkers, and even extract representative spectral features used to characterize and/or classify samples 152. In addition, the integrations of the machine learning approach with SERS can also help us to assign and analyze the multiple spectra. For example, the label-free detection of the gradients of multiple metabolites in the extracellular environment was achieved by the combination of machine learning and SERS optophysiology 79. Artificial intelligence (AI) algorithm has many successful examples in medical imaging. In a recent Nature paper 153, they reported the X-ray data of a breast cancer developed jointly with Google and Deep Mind, which can reduce the false positive rate by 5.7% and false negative rate by 9.4%. Researchers used AI to diagnose the SRS images of brain tumor tissues 154. The AI training model covered 12 common histologic categories and achieved a diagnosis accuracy rate of 94.6%. These developments have fully proved AI technology is powerful in image analysis. We can envision that the SERS united with other advanced algorithms will be a promising and hot trend in cellular and subcellular SERS researches.

## Figures and Tables

**Table 1:** A summary of the experimental methods and results adopted in publications

Category	Methods & Nanosensors	Results	Ref.
Label-free method	The drop-coating Ag NPs on a silicon wafer	The SERS spectra of the cellular surface of the tumor and nontumor cell lines were obtained.	97
	The nuclear targeting AuNRs-based probes	The SERS spectra of the nucleus, malignant and normal epithelial cells were obtained.	98
	The pure AuNPs	The SERS information of the entire mitochondria was obtained.	100
	The mitochondrial targeting AuNRs-based probes and AuNRs	The molecular information of mitochondria from cancer cells *in situ* and *ex situ* method.	120
	The pure AuNRs	The dynamic molecular changes of the living mitochondria upon phototherapy by SERS was investigated.	121
	The silver layered ring substrate	Record the conformation and redox state of the cytochrome c under various conditions.	101
	The Ag@SiO_2_ colloidosomes		122
	The pure AuNPs	Identified and visualized the stages of endocytosis of AuNPs in cells.	103
	The organelles targeting AuNRs-based nanoprobes	The molecular information of different organelles such as nucleus, mitochondria, and lysosome were observed and compared.	104
	The nuclear targeting AuNPs	The real-time SERS monitoring of the molecular dynamics of mitosis in healthy and cancer cells at the molecular level was demonstrated.	24
	The nuclear targeting probes	The SERS spectra of different neuronal cells, and the cytoplasm and nucleus of neuronal cells were obtained.	105
	The SERS-active nanoneedles	Accurately recorded the SERS spectra of the cytoplasm and nucleus.	106
	The nuclear targeting AgNPs	The SERS spectral changes of the nucleus during chemotherapy, PTT, and under the action of ultraviolet light, high oxygen content, and AgNP was studied.	39, 41, 124-127
	The nucleus and mitochondrion-targeting porous Ag/Au with surface decoration of carbon nanodots	The different responses of cancer cells and normal cells upon to PTT	128
	The nucleus and mitochondrion-targeting AuNPs-based probes	Two electrical stimulation modes at organelle level (nucleus and mitochondria) were studied.	130
	The nuclear targeting AuNRs-based probes	The treatment mechanism of Dox and PDT by SERS spectroscopy was reported.	81, 129, 131
SERS label method	The nuclear targeting preloaded Edu in nucleus AuNRs-based probes	The accompanying signal of Edu in the Raman silent range can verify the SERS spectrum of the nucleus.	99
	The organelle-targeting Au@Au core-shell nanoprobes with three different Raman-active molecules into the gap	High-speed, high-resolution, multiplexed subcellular organelle imaging were obtained.	94
	The nuclear and membranous targeting AuNPs with different reporters	FA and LHRH on the surface of the cell membrane were accurately located and detected.	53
	The Raman reporters functionalized nanoprobes	SERS imaging the protein-specific glycosylation on the cell surface	109, 110
	The AgNPs@MPBA nanosensors	SA levels in different cell types and the dynamic expression processes of them were studied.	44, 111
	The nano-tip technology based on SERS	The pH of the nucleus and cytoplasm were detected based on 4-mpy	80
	The organelle targeting AuNRs-based nanoprobes decorated of Mpy	Quantified and monitored of the subcellular ph environment in mitochondria, nucleus, and lysosome	117
	The AuNRs with the decoration of pH-responsive molecules	Changes of lysosomal ph during PTT were revealed.	132
		The intramitochondrial ph evolution, ROS and mitochondrial membrane potential during PDT were revealed.	133
	The Au core-Ag shell nanoparticle (Au@Ag NPs) with 4-MBN in the gap	The intracellular ROS was detected during PTT	134
	The multifunctional nanocarrier based on SERS tags	The intracellular Dox release process was tracked and the killing effect on cancer cells was achieved.	135, 136
	The AuNFs@MBA@RGD with high DOX encapsulation	Efficient intracellular SERS imaging-guided chemo-phototherapy was realized.	137
	The AgNPs@MBN on the ZIF-8@glucose oxidase NPs	The chemo-starvation synergistic treatment of tumor was achieved and the curative effect was got feedback.	138

## Abbreviations

NPs: nanoparticles
SERS: surface-enhanced Raman spectroscopy
ER: endoplasmic reticulum
ATP: adenosine triphosphate
ROS: reactive oxygen species
DNA: deoxyribonucleic acid
MNPs: metal nanoparticles
AuNRs: Au nanorods
GERTs: gap-enhanced Raman tags
MTT: 3-(4,5-dimethylthiazol-2-yl)-2,5-diphenyltetrazolium bromide
WST-1: 2-(4-iodophenyl)-3-(4-nitrophenyl)-5-(2, 4-disulfophenyl)-2H, tetrazolium monosodium salt
PEG: polyethylene glycol
CTAB: cetyltrimethylammonium bromide
BSA: bovine serum albumin
APTMS: aminopropyltrimethoxysilane
SHINs: shell-isolated nanoparticles
HSC: human oral cancer cells
SV: simian virus
Edu: 5-ethynyl-2'-deoxyuridine
AuNPs: Au nanoparticles
PCA-LDA: principal component analysis-linear discriminant analysis
SRS: stimulated Raman scattering
CARS: coherent anti-Stokes Raman scattering
FA: folic acid
LHRH: luteinizing hormone-releasing hormone
SA: sialic acid
EpCAM: epithelial cell adhesion molecule
MPBA: 4-mercaptophenylboronic acid
AgNPs: Ag nanoparticles
NCL: nucleolin
Mpy: 4-mercaptopyridine
PTT: photothermal therapy
PDT: photodynamic therapy
Phe: phenylalanine
Dox: doxorubicin
AuNFs: Au nanoflowers
STORM: stochastic optical reconstruction
MBN: p-mercaptobenzonitrile
TEM: transmission electron microscopy
